# Crystal structure and Hirshfeld surface analyses, inter­molecular inter­action energies and energy frameworks of methyl 6-amino-5-cyano-2-(2-meth­oxy-2-oxoeth­yl)-4-(4-nitro­phen­yl)-4*H*-pyran-3-carboxyl­ate

**DOI:** 10.1107/S2056989025001276

**Published:** 2025-03-11

**Authors:** Farid N. Naghiyev, Tuncer Hökelek, Victor N. Khrustalev, Huseyn M. Mamedov, Alebel N. Belay, Jamshid Ashurov, Ibrahim G. Mamedov

**Affiliations:** a Department of Chemistry, Baku State University, Z. Khalilov Str. 23, Az 1148 Baku, Azerbaijan; b Hacettepe University, Department of Physics, 06800 Beytepe-Ankara, Türkiye; c Peoples’ Friendship University of Russia (RUDN University) Miklukho-Maklay St 6 Moscow 117198 Russian Federation; d N. D. Zelinsky Institute of Organic Chemistry RAS, Leninsky Prosp. 47, Moscow 119991, Russian Federation; e Faculty of Physics, Baku State University, Z. Khalilov Str. 23, Az 1148 Baku, Azerbaijan; f Department of Chemistry, Bahir Dar University, PO Box 79, Bahir Dar, Ethiopia; g Institute of Bioorganic Chemistry, Academy of Sciences of Uzbekistan, M. Ulugbek St. 83, Tashkent, 100125, Uzbekistan; Indian Institute of Science Education and Research Bhopal, India

**Keywords:** crystal structure, carboxyl­ate, hydrogen bond

## Abstract

The title compound, C_17_H_15_N_3_O_7_, contains pyran and phenyl rings, with the pyran ring exhibiting a flattened-boat conformation. In the crystal, inter­molecular N—H⋯N hydrogen bonds link the mol­ecules into centrosymmetric dimers, forming *R*^2^_2_(12) ring motifs. These dimers are linked through N—H⋯O hydrogen bonds into a three-dimensional architecture. A Hirshfeld surface analysis indicates that the most important contributions for the crystal packing are from H⋯O/O⋯H (29.7%), H⋯H (28.7%), H⋯C/C⋯H (16.0%) and H⋯N/N⋯H (12.9%) inter­actions. In addition to van der Waals inter­actions and N—H⋯N and N—H⋯O hydrogen bonds, halogen bonds, tetrel bonds and pnictogen bonds also play an important role in the cohesion of the crystal structure.

## Abstract 2

The title compound contains pyran and phenyl rings, with the pyran ring in a flattened-boat conformation. In the crystal, inter­molecular N—H⋯N hydrogen bonds link the mol­ecules into centrosymmetric dimers, forming *R*^2^_2_(12) ring motifs, which are linked by N—H⋯O hydrogen bonds into a three-dimensional architecture. In addition to van der Waals inter­actions and N—H⋯N and N—H⋯O hydrogen bonds, halogen bonds, tetrel bonds and pnictogen bonds also play an important role in the cohesion of the crystal structure.

## 1. Chemical context

4*H*-Pyrans, a class of heterocyclic compounds containing multifunctional substituents such as nitro (–NO_2_), cyano (–CN), amino (–NH_2_) and ester (–COO*R*) groups have garnered significant inter­est due to their versatile chemical reactivities and wide range of applications (Akkurt *et al.*, 2018 ▸; Askerov *et al.*, 2020 ▸; Khalilov, 2021 ▸). The 4*H*-pyran fragment plays a critical role in pharmaceuticals, agrochemicals, material sciences and catalysis (Mahmoudi *et al.*, 2021 ▸; Gurbanov *et al.*, 2021 ▸). The unique combination of electron-withdrawing (*e.g.* nitro, cyano, ester) and electron-donating (*e.g.* amino) groups imparts remarkable chemical and biological properties, making these mol­ecules indispensable in modern chemistry (Tas *et al.*, 2023 ▸; Khalilov *et al.*, 2024 ▸). Moreover, the structural characteristics of this class of compounds indicate their potential significance in coordination chemistry. The amino group, pyran ring and the biphenyl structure offer multiple coordination sites, enabling the formation of stable metal complexes (Khalilov *et al.*, 2018*a* ▸,*b* ▸; Naghiyev *et al.*, 2021*a* ▸,*b* ▸). Natural products containing 4*H*-pyran derivatives are widespread, forming the core structure of many bioactive compounds. Notable examples include flavonoids (*e.g*. cyanidin, delphinidin), which exhibit anti­oxidant, anti-inflammatory and photoreactive properties (Karimli *et al.*, 2023 ▸; Rzayev & Khalilov, 2024 ▸). These compounds are found in plants, fruits and marine organisms, contributing to diverse biological activities such as anti­microbial, anti­cancer and cardiovascular benefits (Naghiyev *et al.*, 2022 ▸; Mamedov *et al.*, 2020 ▸). Herein, we report the synthesis, mol­ecular and crystal structures and Hirshfeld surface analysis of methyl 6-amino-5- cyano-2-(2-meth­oxy-2-oxoeth­yl)-4-(4-nitro­phen­yl)-4*H*-pyran-3-carboxyl­ate. The results provide comprehensive insights into its mol­ecular geometry, hydrogen-bonding inter­actions and crystal packing, contributing valuable information to the growing database of functionalized carbo- and heterocyclic derivatives.
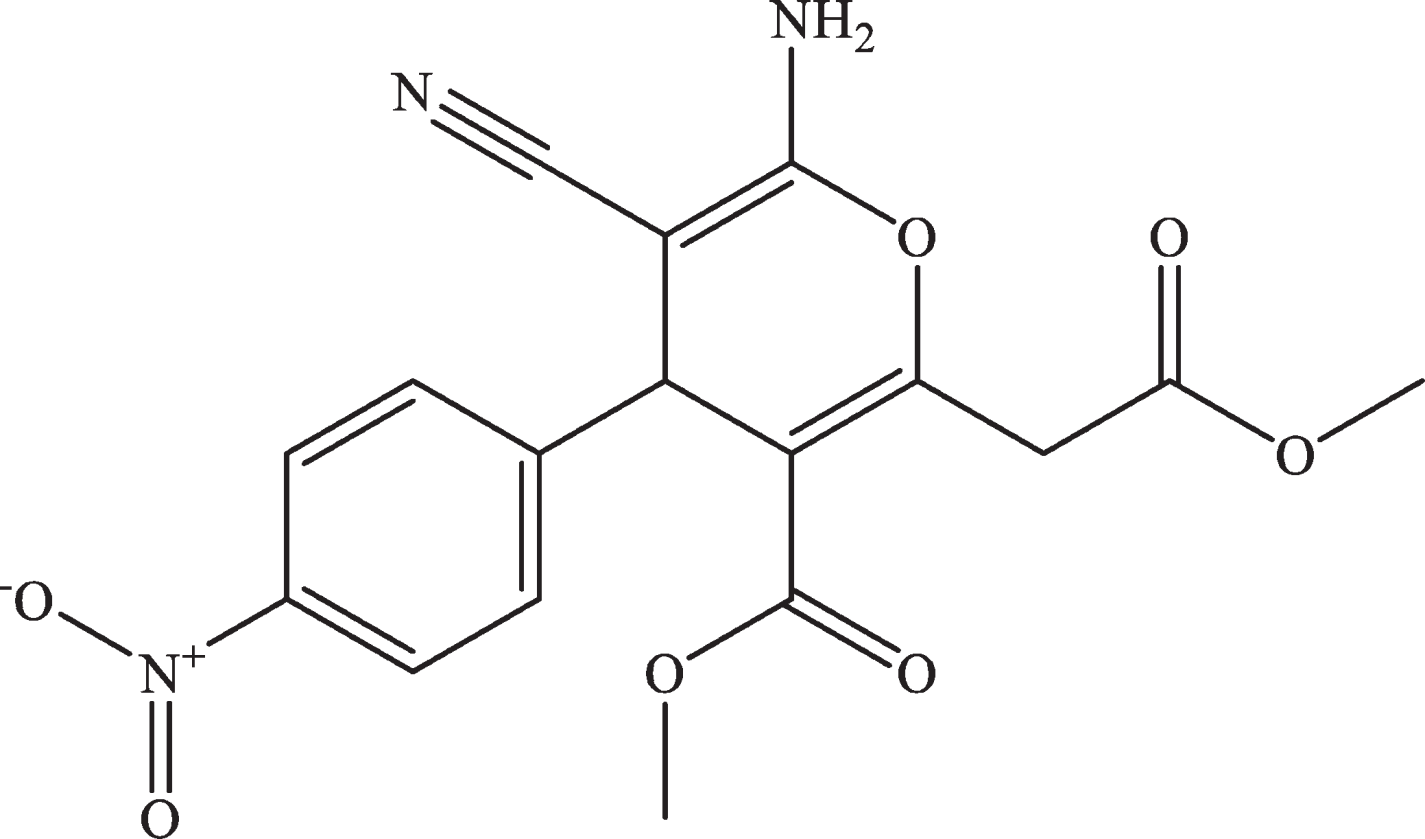


## 2. Structural commentary

**Figure 1:**
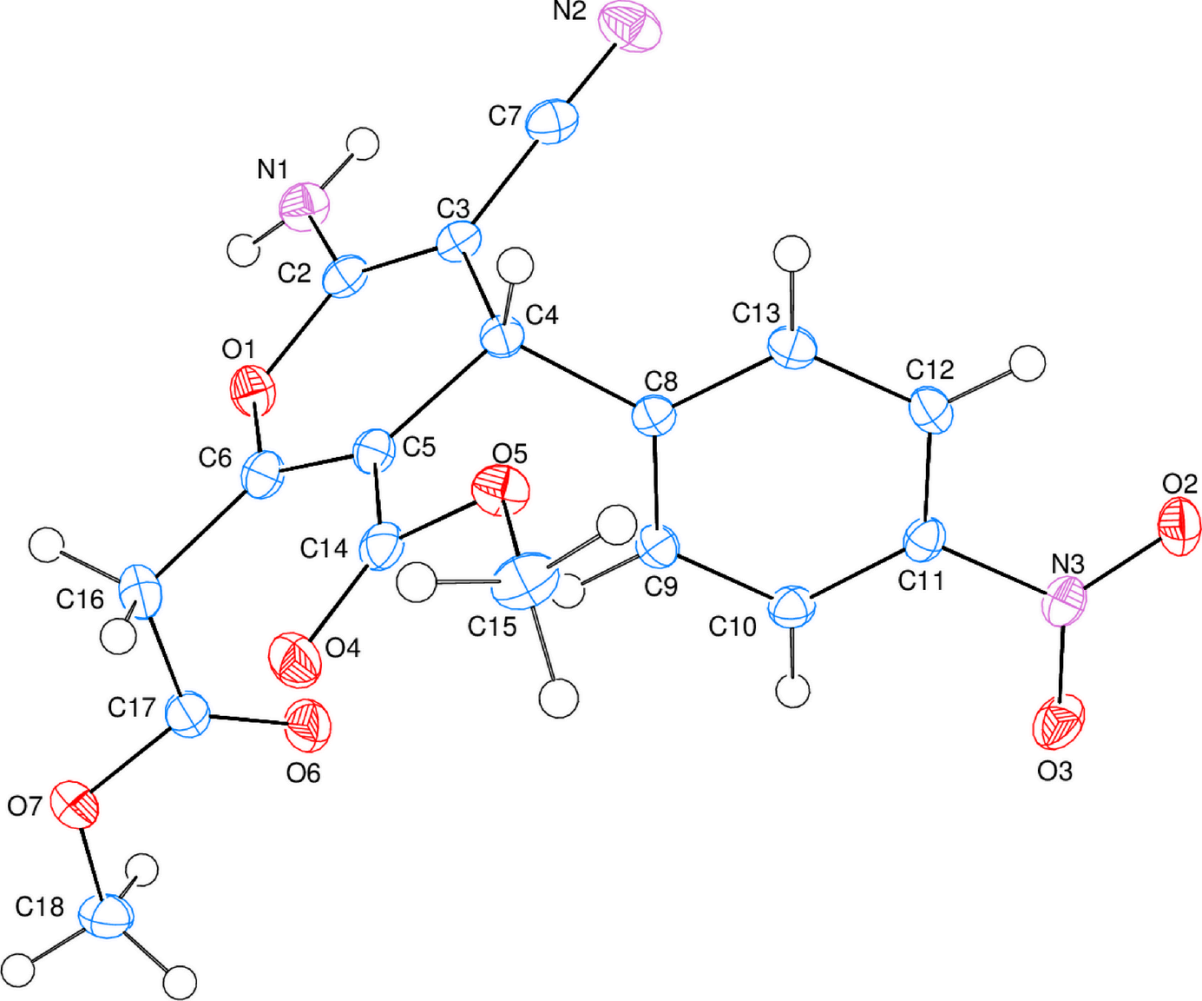
The title mol­ecule with atom-numbering scheme and 50% probability ellipsoids.

**Figure 2:**
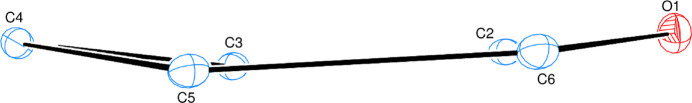
Conformation of the pyran ring (O1/C2–C6).

The title compound contains pyran (*A*:O1/C2–C6) and phenyl (*B*: C8–C13), rings (Fig. 1 ▸). The pyran ring is in a flattened-boat conformation with puckering parameters (Cremer & Pople, 1975 ▸) *Q*_T_ = 0.1228 (10) Å, θ = 110.83 (47)° and φ = 0.7 (5)° (Fig. 2 ▸). Atom N3 is 0.0312 (9) Å away from the best mean plane of the phenyl ring. The nitro (N3/O2/O3) group is oriented at a dihedral angle of 12.06 (8)°with respect to the phenyl ring *B*. The O6—C17—O7 [124.87 (10)°] bond angle in the 2-meth­oxy-2-oxoethyl moiety is larger than the angle O4—C14—O5 [122.65 (9)°] in the carboxyl­ate group. There are no unusual bond lengths or inter­bond angles in the mol­ecule.

## 3. Supra­molecular features

**Figure 3:**
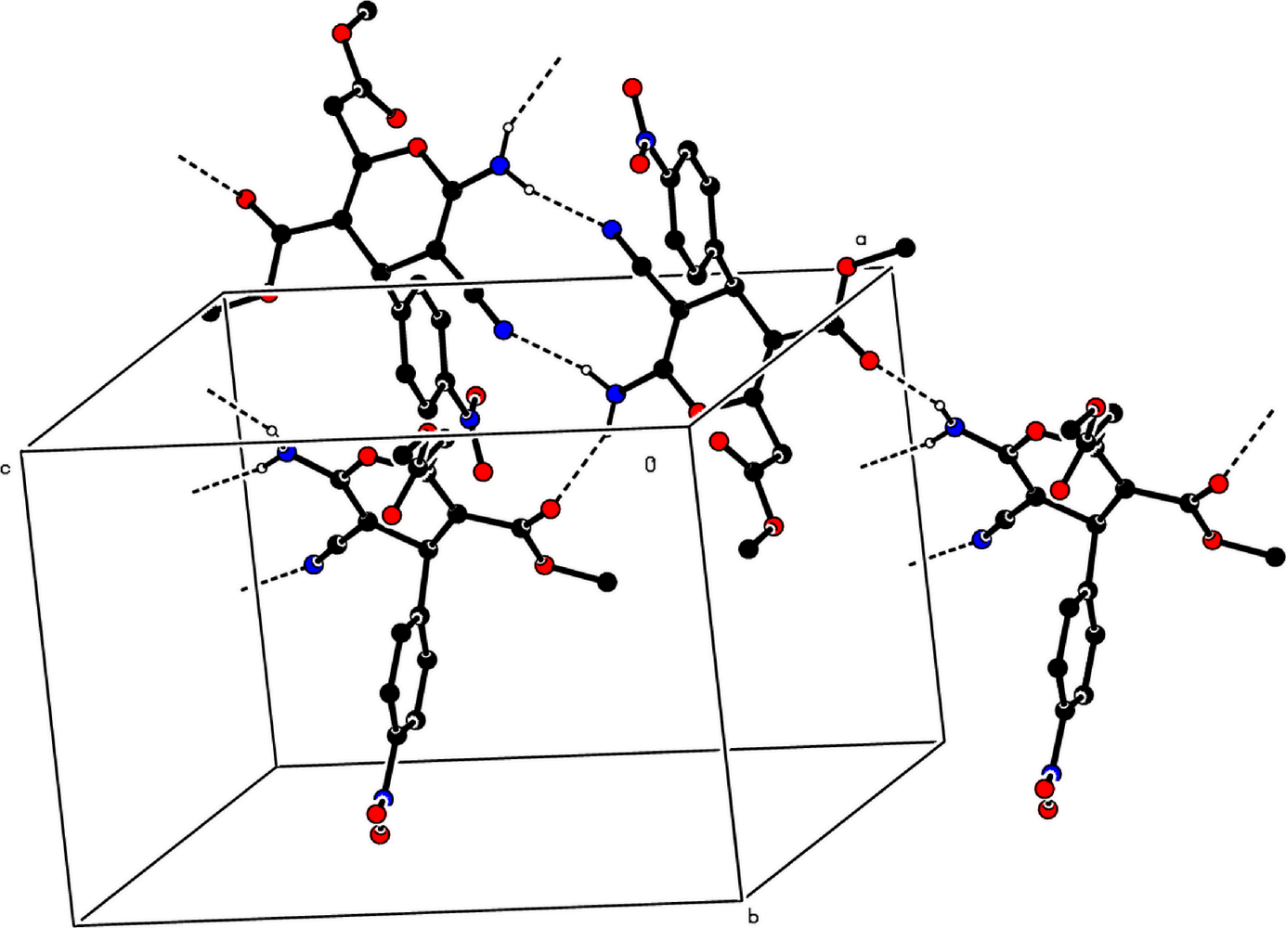
A partial packing diagram. Inter­molecular N—H⋯O and N—H⋯N hydrogen bonds are shown as dashed lines. H atoms not involved in these inter­actions have been omitted for clarity.

**Figure 4:**
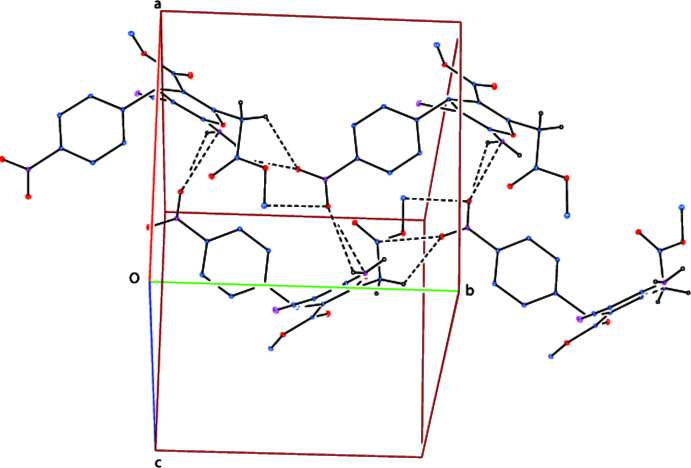
A partial packing diagram showing the halogen, tetrel and pnictogen bonds as dashed lines, where the O, N and C atoms are shown in red, light magenta and blue, respectively, while the H atoms are colourless. H atoms not involved in these inter­actions have been omitted for clarity.

In the crystal, N—H⋯N hydrogen bonds (Table 1 ▸) link the mol­ecules into centrosymmetric dimers,forming 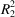
(12) ring motifs (Fig. 3 ▸). These dimers are linked through N—H⋯O hydrogen bonds into a three-dimensional architecture (Fig. 3 ▸). The distance between the N and O atoms may be slightly longer than the sum of the van der Waals radii of the atoms if the chemically involved atoms inter­act electrostatically. In addition, dispersion has an important role in every inter­action. In this case, the nitro oxygen inter­acts above the plane of the –NH_2_ group. These are N—(π hole)⋯O contacts under the category of pnictogen bonding (Varadwaj *et al.*, 2022 ▸). The O3⋯N1, O3⋯H1*B* and O2⋯H16*A* contacts are 3.1495 (13), 3.379 (13) and 2.618 (15) Å, respectively. Tetrel bonding (Varadwaj *et al.*, 2023 ▸) also occurs, involving the nucleophilic oxygen and electrophilic carbon of the carbonyl group [O2⋯C17 = 3.1242 (13), O3⋯C18 = 3.1325 (15) Å. These involve the Burgi–Dunitz trajectory wherein the angular approach of the nucleophile towards an electrophilic centre is probed (Rodríguez *et al.*, 2023 ▸). The Burgi–Dunitz angle herein is 111°. These inter­actions are depicted in Fig. 4 ▸. Thus, in addition to N—H⋯N and N—H⋯O hydrogen bonds, halogen bonds, tetrel bonds and pnictogen bonds also play an important role in the cohesion of the crystal structure. Neither π–π nor C—H⋯π(ring) inter­actions are observed.

## 4. Hirshfeld surface analysis

**Figure 5:**
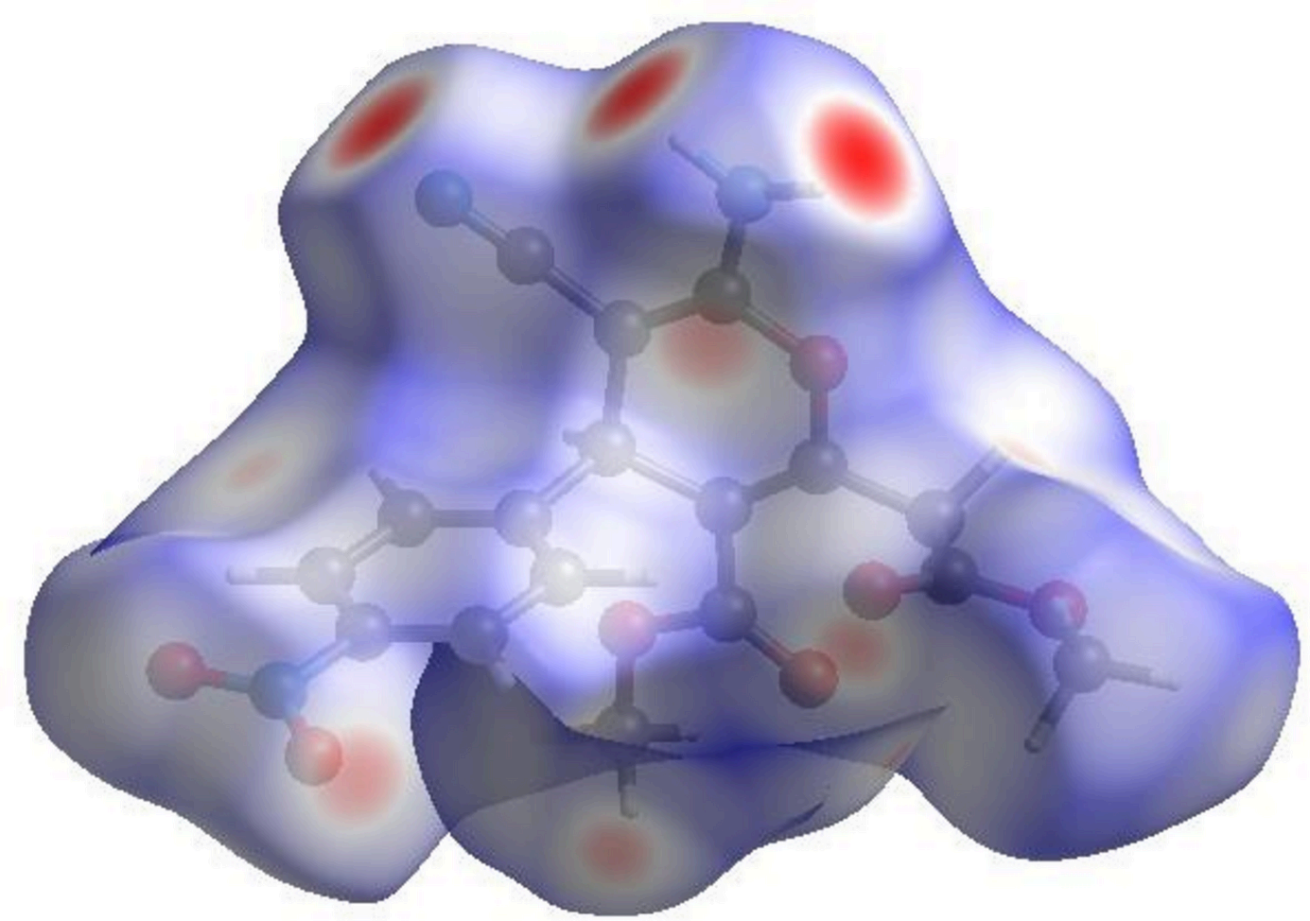
View of the three-dimensional Hirshfeld surface of the title compound plotted over *d*_norm_.

**Figure 6:**
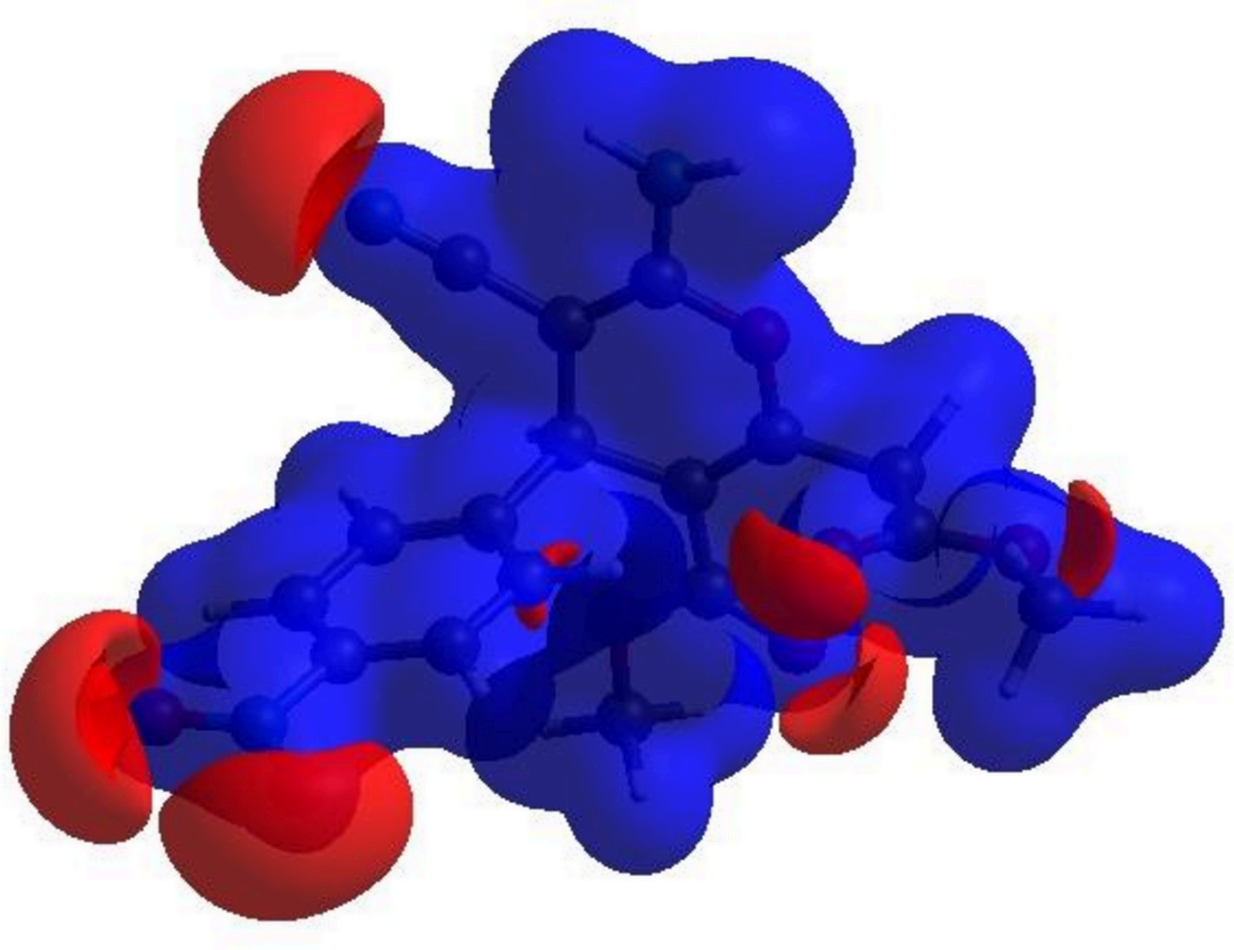
View of the three-dimensional Hirshfeld surface of the title compound plotted over electrostatic potential energy using the STO-3 G basis set at the Hartree–Fock level of theory. Hydrogen-bond donors and acceptors are shown as blue and red regions around the atoms corresponding to positive and negative potentials, respectively.

**Figure 7:**
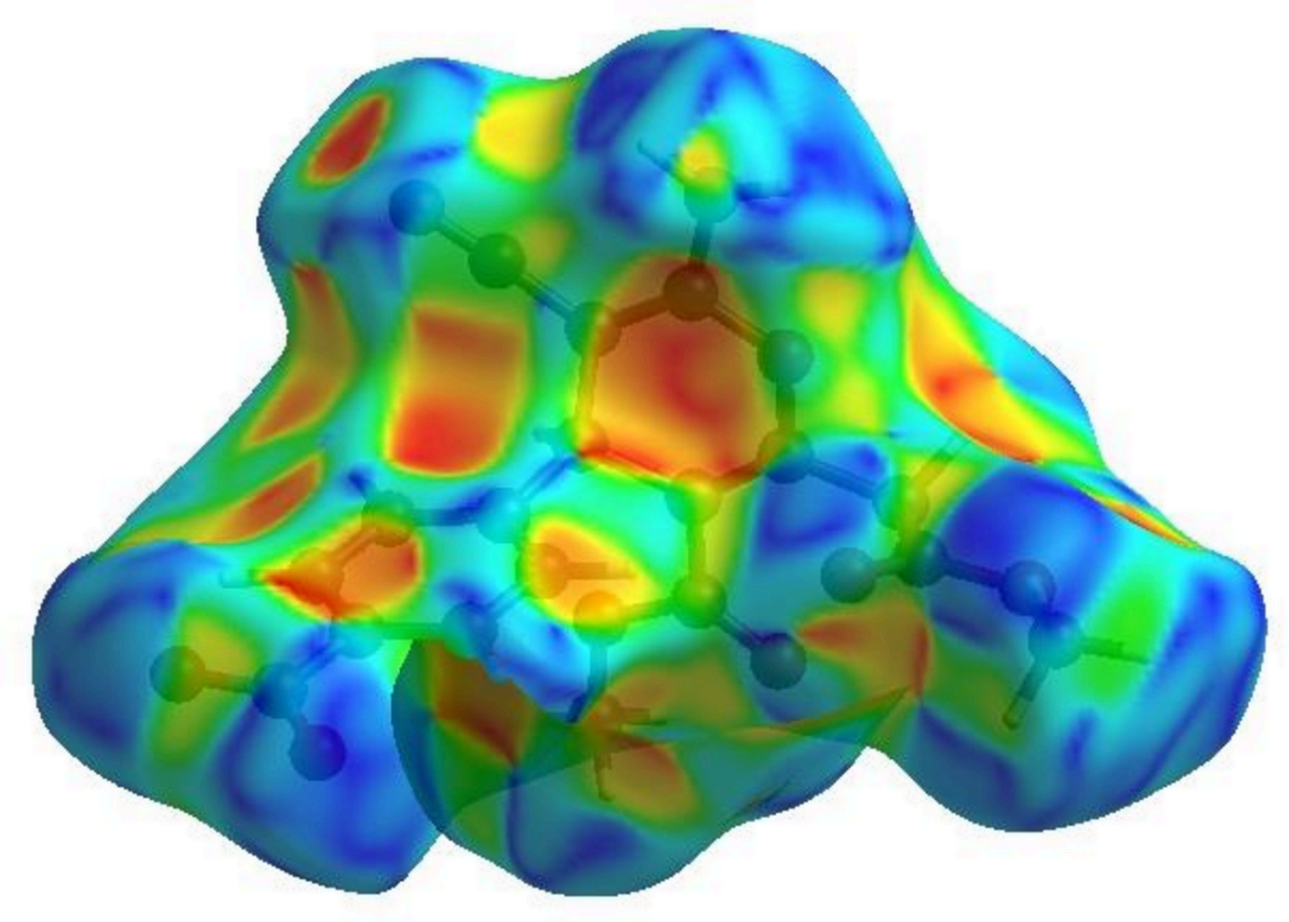
Hirshfeld surface of the title compound plotted over shape-index.

In order to visualize the inter­molecular inter­actions in the title compound, a Hirshfeld surface (HS) analysis (Hirshfeld, 1977 ▸; Spackman & Jayatilaka, 2009 ▸) was carried out using *Crystal Explorer 17.5* (Spackman *et al.*, 2021 ▸). In the HS plotted over *d*_norm_ (Fig. 5 ▸), the white surface indicates contacts with distances equal to the sum of van der Waals radii, and the red and blue colours indicate distances shorter (in close contact) or longer (distinct contact) than the van der Waals radii, respectively (Venkatesan *et al.*, 2016 ▸). The bright-red spots indicate their roles as the respective donors and/or acceptors; they also appear as blue and red regions corresponding to positive and negative potentials on the HS mapped over electrostatic potential (Spackman *et al.*, 2008 ▸; Jayatilaka *et al.*, 2005 ▸) as shown in Fig. 6 ▸. The blue regions indicate the positive electrostatic potential (hydrogen-bond donors), while the red regions indicate the negative electrostatic potential (hydrogen-bond acceptors). The shape-index surface can be used to identify characteristic packing modes, in particular, planar stacking arrangements and the presence of aromatic stacking inter­actions such as C—H⋯π and π–π inter­actions. C—H⋯π inter­actions are seen as red *p*-holes, which are related to the electron ring inter­actions between the CH groups and the centroids of the aromatic rings of neighbouring mol­ecules while π–π interactions are indicated by the presence of adjacent red and blue triangles. Fig. 7 ▸ clearly suggests that there are no C—H⋯π or π–π inter­actions present.

**Figure 8:**
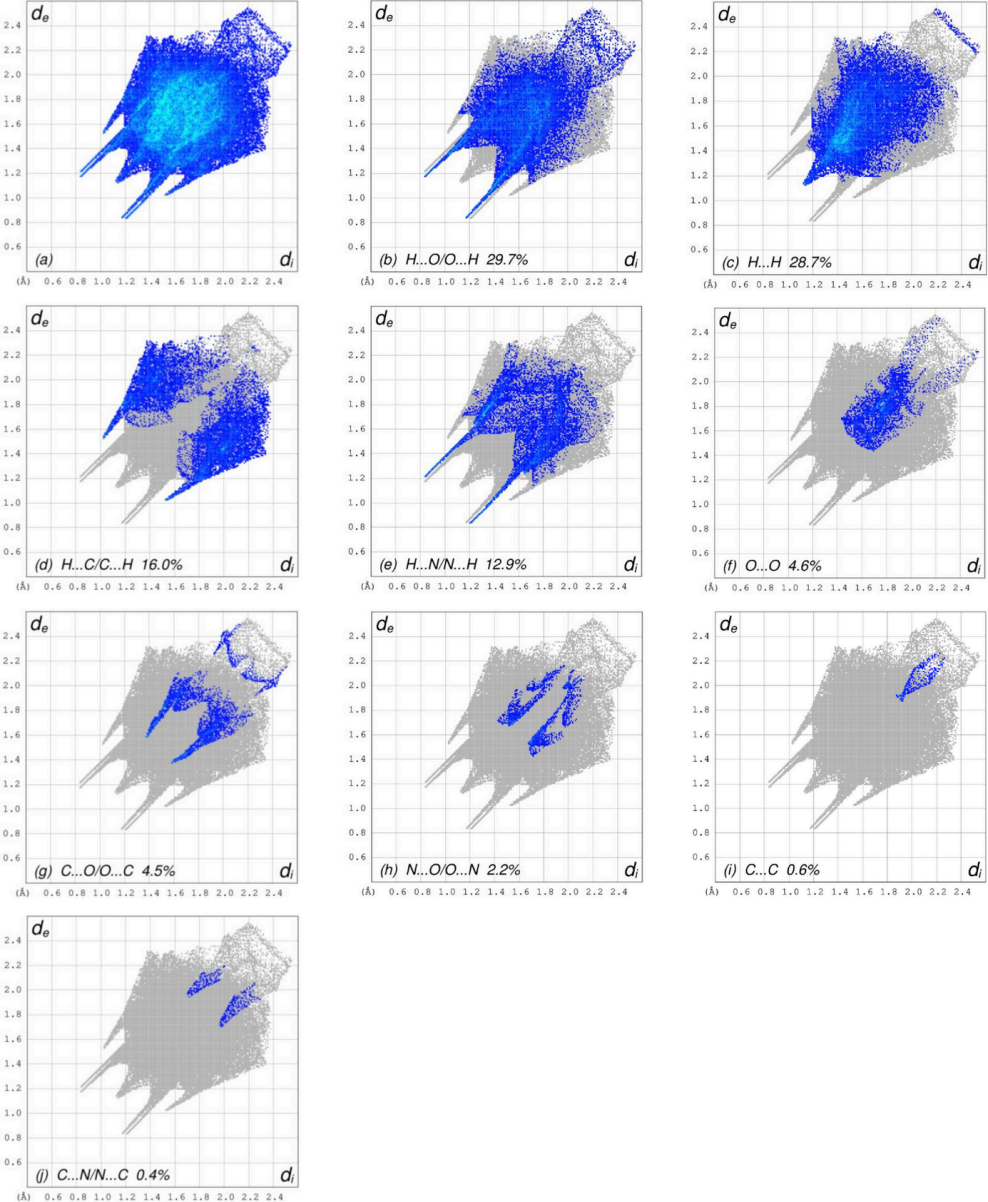
The full two-dimensional fingerprint plots for the title compound, showing (*a*) all inter­actions, and those delineated into (*b*) H⋯O/O⋯H, (*c*) H⋯H, (*d*) H⋯C/C⋯H, (*e*) H⋯N/N⋯H, (*f*) O⋯O, (*g*) C⋯O/O⋯C, (*h*) N⋯O/O⋯N, (i) C⋯C and (*j*) C⋯C/N⋯C inter­actions. The *d*_i_ and *d*_e_ values are the closest inter­nal and external distances (in Å) from given points on the Hirshfeld surface.

The overall two-dimensional fingerprint plot, Fig. 8 ▸*a*, and those delineated into H⋯O/O⋯H, H⋯H, H⋯C/C⋯H, H⋯N/N⋯H, O ⋯ O, C⋯O/O⋯C, N⋯O/O⋯N, C⋯C and C⋯C/N⋯C (McKinnon *et al.*, 2007 ▸) are illustrated in Fig. 8 ▸*b–j*, respectively, together with their relative contributions to the Hirshfeld surface. The most important inter­action is H⋯O/O⋯H (Table 2 ▸) contributing 29.7% to the overall crystal packing, which is shown in Fig. 8 ▸*b* with the pair of spikes at *d*_e_ + *d*_i_ = 2.00 Å. The H⋯H contacts, contributing 28.7% to the overall crystal packing, are represented in Fig. 8 ▸*c* as the widely scattered points of high density due to the large hydrogen content of the mol­ecule with the tip at *d*_e_ = *d*_i_ = 1.12 Å. In the absence of C—H⋯π inter­actions, the characteristic wings of the H⋯C/C⋯H contacts, contributing 16.0% to the overall crystal packing, are shown in Fig. 8 ▸*d* with the tips at *d*_e_ + *d*_i_ = 2.54 Å. The symmetrical pair of spikes of the H⋯N/N⋯H contacts (Fig. 8 ▸*e*; 12.9% contribution to the HS) have the tips at *d*_e_ + *d*_i_ = 2.06 Å. The O⋯O contacts (Fig. 8 ▸*f*) contribute 4.6% to the HS, and they are seen at *d*_e_ = *d*_i_ = 1.54 Å. The C⋯O/O⋯C (Fig. 8 ▸*g*) and N⋯O/O⋯N (Fig. 8 ▸*h*) contacts contribute 4.5% and 2.2%, respectively, to the HS with *d*_e_ + *d*_i_ = 2.96 Å and 3.16 Å, respectively. Finally, the C⋯C (Fig. 8 ▸*i*) and C⋯N/N⋯C (Fig. 8 ▸*j*) contacts with 0.6% and 0.4% contributions to the HS have very low density of points.

The nearest neighbour coordination environment of a mol­ecule can be determined from the colour patches on the HS based on how close to other mol­ecules they are. The Hirshfeld surface representations of contact patches plotted onto the surface are shown for the H⋯O/O⋯H, H⋯H, H⋯C/C⋯H and H⋯ N/N⋯H inter­actions inFig. 9 ▸*a–c*, respectively.

The Hirshfeld surface analysis confirms the importance of H-atom contacts in establishing the packing. The large number of H⋯O/O⋯H, H⋯H, H⋯ C/C⋯H and H⋯N/N⋯H inter­actions suggest that van der Waals inter­actions and hydrogen bonding play the major roles in the crystal packing (Hathwar *et al.*, 2015 ▸).

## 5. Crystal voids

**Figure 10:**
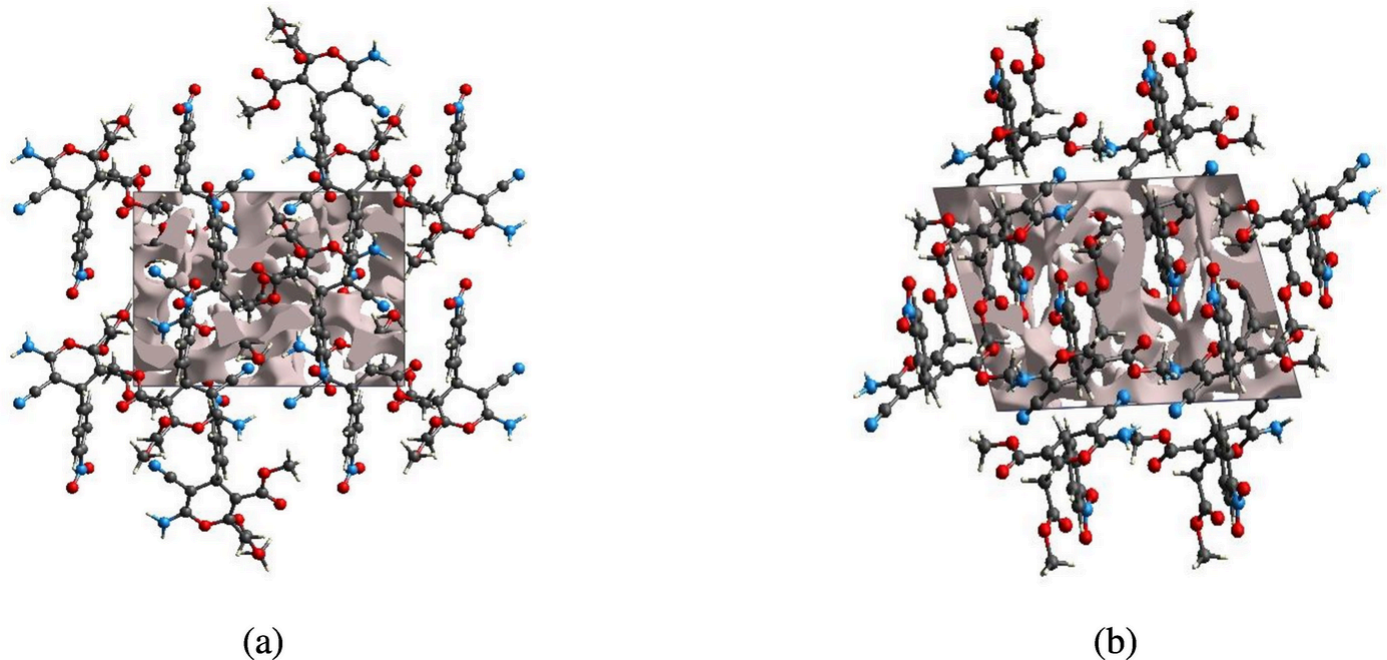
Graphical views of voids in the crystal packing of the title compound (*a*) along the *a*-axis and (*b*) along the *b*-axis directions.

The strength of the crystal packing is important for determining the response to an applied mechanical force. If the crystal packing results in significant voids, then the mol­ecules are not tightly packed and a small amount of applied external mechanical force may easily break the crystal. For checking the mechanical stability of the crystal, a void analysis was performed by adding up the electron densities of the spherically symmetric atoms contained in the asymmetric unit (Turner *et al.*, 2011 ▸). The void surface is defined as an isosurface of the procrystal electron density and is calculated for the whole unit cell where the void surface meets the boundary of the unit cell and capping faces are generated to create an enclosed volume. The volume of the crystal voids (Fig. 10 ▸*a*,*b*) and the percentage of free space in the unit cell are calculated as 170.52 Å^3^ and 10.20%, respectively. Thus, the crystal packing appears compact and the mechanical stability should be substantial.

## 6. Inter­action energy calculations and energy frameworks

**Figure 11:**
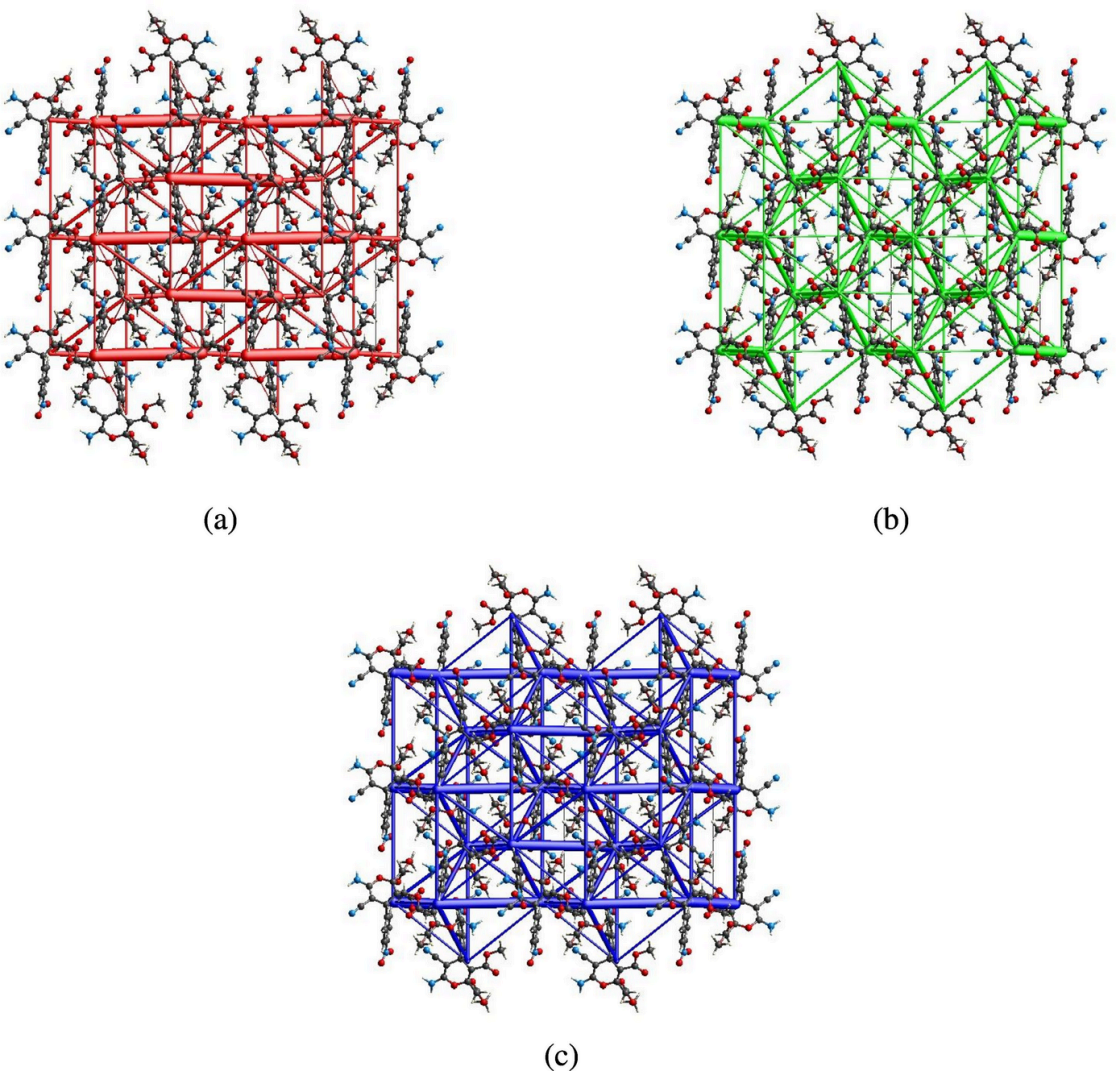
The energy frameworks for a cluster of mol­ecules of the title compound viewed down the *a*-axis showing the (*a*) electrostatic energy, (*b*) dispersion energy and (*c*) total energy diagrams. The cylindrical radius is proportional to the relative strength of the corresponding energies and they were adjusted to the same scale factor of 80 with cut-off value of 5 kJ mol^−1^ within 2×2×2 unit cells.

The inter­molecular inter­action energies were calculated using the CE–B3LYP/6–31G(d,p) energy model available in *Crystal Explorer 17.5* (Spackman *et al.*, 2021 ▸), where a cluster of mol­ecules is generated by applying crystallographic symmetry operations with respect to a selected central mol­ecule within the radius of 3.8 Å by default (Turner *et al.*, 2014 ▸). The total inter­molecular energy (*E*_tot_) is the sum of the electrostatic (*E*_ele_), polarization (*E*_pol_), dispersion (*E*_dis_) and exchange-repulsion (*E*_rep_) energies (Turner *et al.*, 2015 ▸) with scale factors of 1.057, 0.740, 0.871 and 0.618, respectively (Mackenzie *et al.*, 2017 ▸). Hydrogen-bonding inter­action energies (in kJ mol^−1^) were calculated to be −29.7 (*E*_ele_), −7.0 (*E*_pol_), −74.4 (*E*_dis_), 55.0 (*E*_rep_) and −67.3 (*E*_tot_) for N1—H1*A*⋯O4 and −71.4 (*E*_ele_), −15.8 (*E*_pol_), −13.0 (*E*_dis_), 58.0 (*E*_rep_) and −62.6 (*E*_tot_) for N1—H11*B*⋯N2. Energy frameworks combine the calculation of inter­molecular inter­action energies with a graphical representation of their magnitude (Turner *et al.*, 2015 ▸). Energies between mol­ecular pairs are represented as cylinders joining the centroids of pairs of mol­ecules with the cylinder radius proportional to the relative strength of the corresponding inter­action energy. Energy frameworks were constructed for *E*_ele_ (red cylinders), *E*_dis_ (green cylinders) and *E*_tot_ (blue cylinders) (Fig. 11 ▸*a*,*b*,*c*). The evaluation of the electrostatic, dispersion and total energy frameworks indicates that the stabilization is dominated by the electrostatic energy contributions in the crystal structure of the title compound.

## 7. Synthesis and crystallization

The title compound was synthesized following a reported procedure (Heber & Stoyanov, 2003 ▸). A mixture of 1.0 g (0.0051 mol) of *p*-nitro­benzyl­idenemalono­nitrile and 0.9 g (0.0052 mol) of dimethyl-1.3-acetonedi­carboxyl­ate was dissolved in 30 ml of methyl alcohol with stirring for 20 min, 3–4 drops of methyl­piperazine were added and stirring was continued. The reaction mixture was kept for 48 h. After that, crystals precipitated as the solvent evaporated. Colorless crystals were obtained from an ethanol/water (3:1 *v*/*v*) solution after recrystallization. Yield 75.70%, m.p. 449–450 K. ^1^H NMR (300 MHz, DMSO-*d*_6_, δ). 3.48 (*s*, 3H, OCH_3_), 3.69 (*s*, 3H, OCH_3_), 3.74 (*d*, 1H, CH_2_, *J* = 17.1 Hz), 3.96 (*d*, 1H, CH_2_, *J* = 17.1 Hz), 4.52 (*s*, 1H, PhCH), 7.11 (*s*, 2H, NH_2_), 7.47 (*d*, 2H, arom., *J* = 8.4 Hz), 8.21 (*d*, 2H, arom., *J* = 8.4 Hz).

## 8. Refinement

Crystal data, data collection and structure refinement details are summarized in Table 3 ▸. The NH hydrogen atoms were located in a difference-Fourier map, and refined freely. The C-bound hydrogen-atom positions were calculated geometrically at distances of 1.00 Å (for methine CH), 0.95 Å (for aromatic CH), 0.99 Å (for CH_2_) and 0.98 Å (for CH_3_) and refined using a riding model by applying the constraint *U*_iso_(H) = *k U*_eq_ (C), where *k* = 1.5 for methyl H atoms and *k* = 1.2 for all other C-bound H atoms.

## Supplementary Material

Crystal structure: contains datablock(s) I. DOI: 10.1107/S2056989025001276/dx2064sup1.cif

Structure factors: contains datablock(s) I. DOI: 10.1107/S2056989025001276/dx2064Isup2.hkl

Supporting information file. DOI: 10.1107/S2056989025001276/dx2064Isup3.cml

CCDC reference: 2423124

Additional supporting information:  crystallographic information; 3D view; checkCIF report

## Figures and Tables

**Figure 9:**
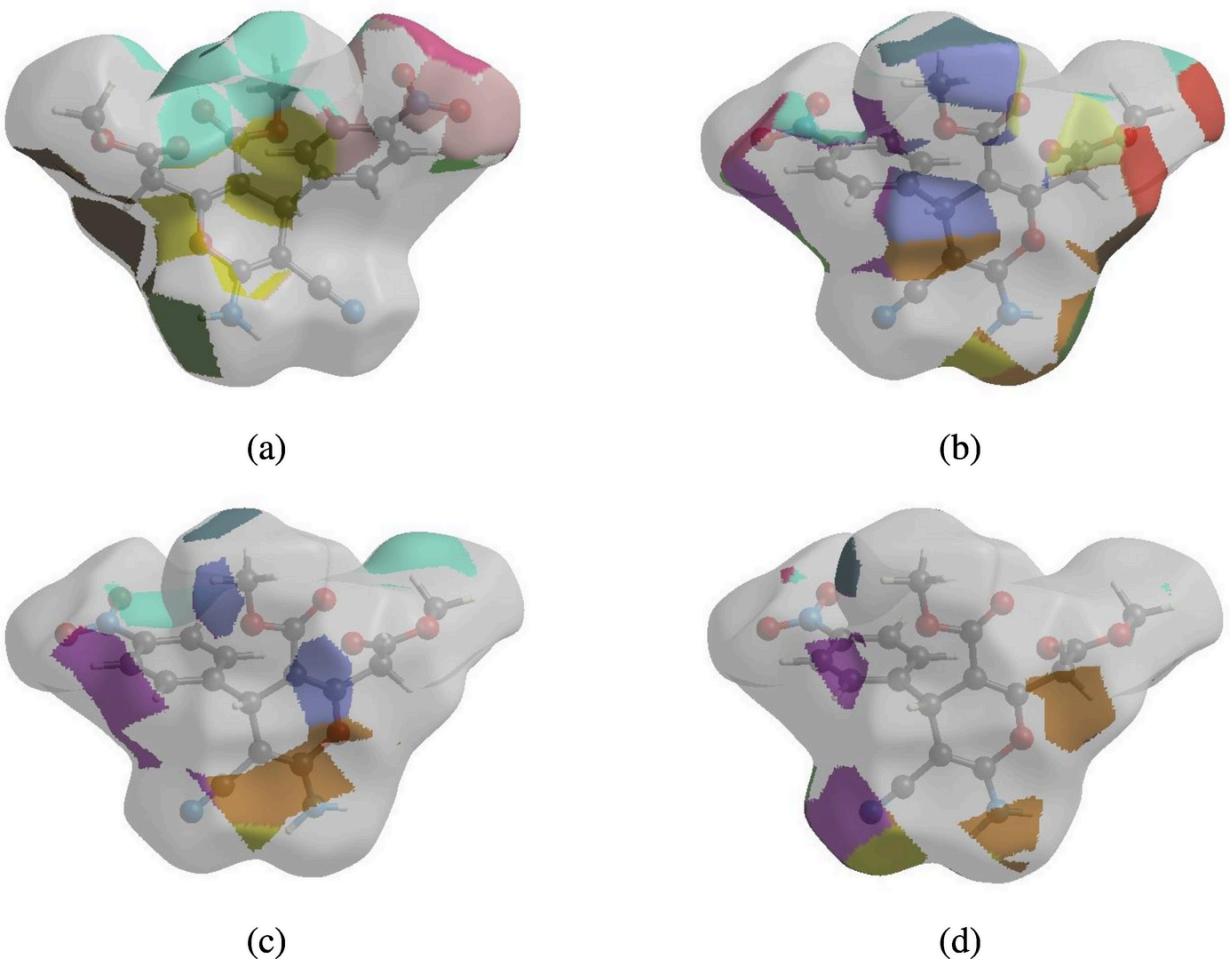
The Hirshfeld surface representations of contact patches plotted onto the surface for (*a*) H⋯O/O⋯H, (*b*) H⋯H, (*c*) H⋯C/C⋯H and (*d*) H⋯N/N⋯H inter­actions.

**Table 1:** Hydrogen-bond geometry (Å, °)

*D*—H⋯*A*	*D*—H	H⋯*A*	*D*⋯*A*	*D*—H⋯*A*
N1—H1*A*⋯O4^ii^	0.887 (16)	2.112 (16)	2.9362 (12)	154.1 (13)
N1—H1*B*⋯N2^iv^	0.892 (17)	2.152 (17)	3.0283 (14)	167.2 (14)

Symmetry codes: (ii) 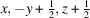; (iv) 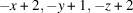.

**Table 2:** Selected interatomic distances (Å)

C17⋯O2^i^	3.1242 (13)	O4⋯H15*C*	2.64
C18⋯O3^i^	3.1325 (15)	O5⋯H4	2.41
O4⋯C16	2.8924 (13)	H15*C*⋯O6^iii^	2.47
N1⋯O4^ii^	2.9361 (13)	O6⋯H18*A*	2.66
O5⋯C8	2.9834 (12)	O6⋯H18*B*	2.58
O2⋯H12	2.44	N2⋯N1^iv^	3.0282 (14)
H16*A*⋯O2^i^	2.62	H1*B*⋯N2^iv^	2.152 (16)
O3⋯H10	2.4456	C7⋯C13	3.3501 (15)
H10⋯O4^iii^	2.57	C5⋯H9	2.67
O4⋯H16*B*	2.20	C7⋯H1*B*	2.616 (16)
H1*A*⋯O4^ii^	2.112 (16)	C14⋯H16*B*	2.70
O4⋯H15*A*	2.54	H4⋯H13	2.36

Symmetry codes: (i) 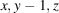; (ii) 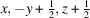; (iii) 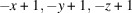; (iv) 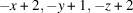.

**Table 3:** Experimental details

Crystal data	
Chemical formula	C_17_H_15_N_3_O_7_
*M*_r_	373.32
Crystal system, space group	Monoclinic, *P*2_1_/*c*
Temperature (K)	100
*a*, *b*, *c* (Å)	10.27958 (5), 11.18770 (6), 15.09983 (8)
β (°)	105.6759 (5)
*V* (Å^3^)	1671.96 (2)
*Z*	4
Radiation type	Cu *K*α
μ (mm^−1^)	1.00
Crystal size (mm)	0.21 × 0.15 × 0.13

Data collection	
Diffractometer	XtaLAB Synergy-i CCD diffractometer
Absorption correction	Gaussian (*CrysAlis PRO*; Rigaku OD, 2022 ▸)
*T*_min_, *T*_max_	0.399, 1.000
No. of measured, independent and observed [*I* > 2σ(*I*)] reflections	43458, 3550, 3505
*R*_int_	0.024
(sin θ/λ)_max_ (Å^−1^)	0.634

Refinement	
*R*[*F*^2^ > 2σ(*F*^2^)], *wR*(*F*^2^), *S*	0.031, 0.082, 1.05
No. of reflections	3550
No. of parameters	254
H-atom treatment	H atoms treated by a mixture of independent and constrained refinement
Δρ_max_, Δρ_min_ (e Å^−3^)	0.25, −0.26

Computer programs: *CrysAlis PRO* (Rigaku OD, 2022 ▸), *SHELXT2014/5* (Sheldrick, 2015*a* ▸), *SHELXL2018/3* (Sheldrick, 2015*b* ▸) and *SHELXTL* (Sheldrick, 2008 ▸).

## supplementary crystallographic information

### Methyl 6-amino-5-cyano-2-(2-methoxy-2-oxoethyl)-4-(4-nitrophenyl)-4H-pyran-3-carboxylate Crystal data

**Table:**

C_17_H_15_N_3_O_7_	*F*(000) = 776
*M**_r_* = 373.32	*D*_x_ = 1.483 Mg m^−^^3^
Monoclinic, *P*2_1_/*c*	Cu *K*α radiation, λ = 1.54184 Å
*a* = 10.27958 (5) Å	Cell parameters from 36303 reflections
*b* = 11.18770 (6) Å	θ = 4.0–77.7°
*c* = 15.09983 (8) Å	µ = 1.00 mm^−^^1^
β = 105.6759 (5)°	*T* = 100 K
*V* = 1671.96 (2) Å^3^	Prism, colourless
*Z* = 4	0.21 × 0.15 × 0.13 mm

### Methyl 6-amino-5-cyano-2-(2-methoxy-2-oxoethyl)-4-(4-nitrophenyl)-4H-pyran-3-carboxylate Data collection

**Table:**

XtaLAB Synergy-i CCD diffractometer	3505 reflections with *I* > 2σ(*I*)
Radiation source: micro-focus sealed X-ray tube	*R*_int_ = 0.024
φ and ω scans	θ_max_ = 77.8°, θ_min_ = 4.5°
Absorption correction: gaussian (CrysAlisPro; Rigaku OD, 2022)	*h* = −13→13
*T*_min_ = 0.399, *T*_max_ = 1.000	*k* = −14→14
43458 measured reflections	*l* = −19→18
3550 independent reflections	

### Methyl 6-amino-5-cyano-2-(2-methoxy-2-oxoethyl)-4-(4-nitrophenyl)-4H-pyran-3-carboxylate Refinement

**Table:**

Refinement on *F*^2^	Primary atom site location: difference Fourier map
Least-squares matrix: full	Secondary atom site location: difference Fourier map
*R*[*F*^2^ > 2σ(*F*^2^)] = 0.031	Hydrogen site location: mixed
*wR*(*F*^2^) = 0.082	H atoms treated by a mixture of independent and constrained refinement
*S* = 1.05	*w* = 1/[σ^2^(*F*_o_^2^) + (0.0426*P*)^2^ + 0.66*P*] where *P* = (*F*_o_^2^ + 2*F*_c_^2^)/3
3550 reflections	(Δ/σ)_max_ = 0.001
254 parameters	Δρ_max_ = 0.25 e Å^−^^3^
0 restraints	Δρ_min_ = −0.26 e Å^−^^3^

### Methyl 6-amino-5-cyano-2-(2-methoxy-2-oxoethyl)-4-(4-nitrophenyl)-4H-pyran-3-carboxylate Special details

**Table:**

Geometry. All esds (except the esd in the dihedral angle between two l.s. planes) are estimated using the full covariance matrix. The cell esds are taken into account individually in the estimation of esds in distances, angles and torsion angles; correlations between esds in cell parameters are only used when they are defined by crystal symmetry. An approximate (isotropic) treatment of cell esds is used for estimating esds involving l.s. planes.

### Methyl 6-amino-5-cyano-2-(2-methoxy-2-oxoethyl)-4-(4-nitrophenyl)-4H-pyran-3-carboxylate Fractional atomic coordinates and isotropic or equivalent isotropic displacement parameters (Å^2^)

**Table:**

	*x*	*y*	*z*	*U*_iso_*/*U*_eq_	
O1	0.77251 (7)	0.28311 (6)	0.73783 (5)	0.01923 (16)	
O2	0.60222 (8)	1.02372 (7)	0.72626 (6)	0.02412 (17)	
O3	0.41912 (8)	0.92231 (7)	0.66787 (6)	0.02586 (18)	
O4	0.72560 (8)	0.39316 (7)	0.45860 (5)	0.02369 (17)	
O5	0.85282 (7)	0.55117 (7)	0.51861 (5)	0.01883 (16)	
O6	0.47708 (8)	0.31475 (7)	0.61457 (6)	0.02503 (18)	
O7	0.45768 (8)	0.13799 (7)	0.54080 (5)	0.02177 (17)	
N1	0.86641 (9)	0.29814 (9)	0.88857 (7)	0.02135 (19)	
H1A	0.8151 (15)	0.2350 (14)	0.8908 (10)	0.028 (4)*	
H1B	0.9009 (16)	0.3401 (14)	0.9398 (11)	0.033 (4)*	
N2	1.03545 (10)	0.58976 (9)	0.92255 (7)	0.0275 (2)	
N3	0.54251 (9)	0.93095 (8)	0.69545 (6)	0.01857 (18)	
C2	0.84714 (10)	0.35325 (9)	0.80715 (7)	0.0170 (2)	
C3	0.89769 (10)	0.46004 (9)	0.78966 (7)	0.0160 (2)	
C4	0.86693 (9)	0.51344 (9)	0.69395 (6)	0.01460 (19)	
H4	0.9540	0.5385	0.6820	0.018*	
C5	0.80138 (9)	0.42006 (9)	0.62345 (7)	0.0156 (2)	
C6	0.75493 (10)	0.31621 (9)	0.64726 (7)	0.0170 (2)	
C7	0.97348 (10)	0.52994 (10)	0.86393 (7)	0.0189 (2)	
C8	0.77799 (9)	0.62376 (9)	0.68853 (6)	0.01387 (19)	
C9	0.63820 (10)	0.61374 (9)	0.67295 (7)	0.0169 (2)	
H9	0.5963	0.5375	0.6612	0.020*	
C10	0.56015 (10)	0.71444 (9)	0.67456 (7)	0.0175 (2)	
H10	0.4650	0.7082	0.6641	0.021*	
C11	0.62397 (10)	0.82428 (9)	0.69179 (6)	0.01549 (19)	
C12	0.76225 (10)	0.83757 (9)	0.70665 (7)	0.0172 (2)	
H12	0.8035	0.9141	0.7179	0.021*	
C13	0.83852 (10)	0.73601 (9)	0.70462 (7)	0.0169 (2)	
H13	0.9335	0.7430	0.7143	0.020*	
C14	0.78635 (10)	0.45024 (9)	0.52536 (7)	0.0164 (2)	
C15	0.84512 (11)	0.58914 (11)	0.42552 (7)	0.0219 (2)	
H15A	0.8778	0.5248	0.3931	0.033*	
H15B	0.9012	0.6604	0.4274	0.033*	
H15C	0.7511	0.6079	0.3932	0.033*	
C16	0.67455 (11)	0.22033 (9)	0.58768 (7)	0.0198 (2)	
H16A	0.7080	0.1409	0.6126	0.024*	
H16B	0.6859	0.2267	0.5248	0.024*	
C17	0.52626 (10)	0.23216 (9)	0.58392 (7)	0.0182 (2)	
C18	0.31474 (11)	0.13772 (11)	0.53517 (8)	0.0258 (2)	
H18A	0.2708	0.2054	0.4976	0.039*	
H18B	0.3025	0.1447	0.5971	0.039*	
H18C	0.2742	0.0629	0.5070	0.039*	

### Methyl 6-amino-5-cyano-2-(2-methoxy-2-oxoethyl)-4-(4-nitrophenyl)-4H-pyran-3-carboxylate Atomic displacement parameters (Å^2^)

**Table:**

	*U*^11^	*U*^22^	*U*^33^	*U*^12^	*U*^13^	*U*^23^
O1	0.0228 (4)	0.0168 (3)	0.0193 (4)	−0.0020 (3)	0.0077 (3)	0.0004 (3)
O2	0.0298 (4)	0.0153 (4)	0.0275 (4)	0.0010 (3)	0.0082 (3)	−0.0020 (3)
O3	0.0180 (4)	0.0233 (4)	0.0373 (5)	0.0047 (3)	0.0092 (3)	0.0058 (3)
O4	0.0295 (4)	0.0212 (4)	0.0180 (4)	0.0001 (3)	0.0025 (3)	−0.0042 (3)
O5	0.0197 (4)	0.0225 (4)	0.0147 (3)	−0.0020 (3)	0.0053 (3)	0.0017 (3)
O6	0.0215 (4)	0.0221 (4)	0.0314 (4)	0.0010 (3)	0.0070 (3)	−0.0063 (3)
O7	0.0218 (4)	0.0199 (4)	0.0220 (4)	−0.0033 (3)	0.0032 (3)	−0.0035 (3)
N1	0.0228 (4)	0.0213 (5)	0.0201 (5)	−0.0002 (4)	0.0062 (4)	0.0054 (4)
N2	0.0292 (5)	0.0317 (5)	0.0189 (4)	−0.0087 (4)	0.0016 (4)	0.0029 (4)
N3	0.0213 (4)	0.0167 (4)	0.0188 (4)	0.0024 (3)	0.0074 (3)	0.0033 (3)
C2	0.0141 (4)	0.0191 (5)	0.0185 (5)	0.0036 (4)	0.0056 (4)	0.0012 (4)
C3	0.0141 (4)	0.0176 (5)	0.0160 (5)	0.0025 (4)	0.0036 (4)	0.0014 (4)
C4	0.0136 (4)	0.0154 (5)	0.0151 (4)	−0.0001 (3)	0.0046 (3)	0.0002 (3)
C5	0.0145 (4)	0.0158 (5)	0.0170 (5)	0.0023 (3)	0.0052 (4)	−0.0016 (4)
C6	0.0167 (4)	0.0172 (5)	0.0185 (5)	0.0026 (4)	0.0072 (4)	−0.0009 (4)
C7	0.0173 (5)	0.0225 (5)	0.0169 (5)	0.0005 (4)	0.0044 (4)	0.0056 (4)
C8	0.0155 (4)	0.0157 (5)	0.0105 (4)	0.0002 (3)	0.0036 (3)	0.0008 (3)
C9	0.0161 (5)	0.0155 (5)	0.0189 (5)	−0.0020 (4)	0.0041 (4)	0.0002 (4)
C10	0.0141 (4)	0.0189 (5)	0.0191 (5)	−0.0004 (4)	0.0037 (4)	0.0017 (4)
C11	0.0183 (5)	0.0151 (5)	0.0133 (4)	0.0026 (4)	0.0047 (3)	0.0016 (3)
C12	0.0194 (5)	0.0151 (5)	0.0171 (5)	−0.0034 (4)	0.0047 (4)	−0.0012 (4)
C13	0.0141 (4)	0.0188 (5)	0.0175 (5)	−0.0022 (4)	0.0041 (4)	−0.0006 (4)
C14	0.0154 (4)	0.0163 (5)	0.0179 (5)	0.0040 (4)	0.0055 (4)	−0.0009 (4)
C15	0.0216 (5)	0.0294 (6)	0.0159 (5)	0.0037 (4)	0.0068 (4)	0.0058 (4)
C16	0.0218 (5)	0.0159 (5)	0.0240 (5)	−0.0021 (4)	0.0098 (4)	−0.0038 (4)
C17	0.0218 (5)	0.0166 (5)	0.0156 (5)	−0.0010 (4)	0.0042 (4)	0.0007 (4)
C18	0.0197 (5)	0.0261 (6)	0.0274 (6)	−0.0027 (4)	−0.0006 (4)	−0.0002 (4)

### Methyl 6-amino-5-cyano-2-(2-methoxy-2-oxoethyl)-4-(4-nitrophenyl)-4H-pyran-3-carboxylate Geometric parameters (Å, º)

**Table:**

O1—C2	1.3661 (13)	C5—C14	1.4860 (14)
O1—C6	1.3807 (12)	C6—C16	1.4970 (14)
O2—N3	1.2313 (12)	C8—C13	1.3934 (14)
O3—N3	1.2273 (12)	C8—C9	1.3965 (13)
O4—C14	1.2139 (13)	C9—C10	1.3870 (14)
O5—C14	1.3378 (13)	C9—H9	0.9500
O5—C15	1.4501 (12)	C10—C11	1.3842 (14)
O6—C17	1.2042 (13)	C10—H10	0.9500
O7—C17	1.3353 (12)	C11—C12	1.3858 (14)
O7—C18	1.4487 (13)	C12—C13	1.3855 (14)
N1—C2	1.3413 (14)	C12—H12	0.9500
N1—H1A	0.887 (16)	C13—H13	0.9500
N1—H1B	0.892 (17)	C15—H15A	0.9800
N2—C7	1.1543 (15)	C15—H15B	0.9800
N3—C11	1.4674 (13)	C15—H15C	0.9800
C2—C3	1.3570 (14)	C16—C17	1.5157 (14)
C3—C7	1.4156 (14)	C16—H16A	0.9900
C3—C4	1.5162 (13)	C16—H16B	0.9900
C4—C5	1.5137 (13)	C18—H18A	0.9800
C4—C8	1.5250 (13)	C18—H18B	0.9800
C4—H4	1.0000	C18—H18C	0.9800
C5—C6	1.3416 (14)		
			
C17···O2^i^	3.1242 (13)	O4···H15C	2.64
C18···O3^i^	3.1325 (15)	O5···H4	2.41
O4···C16	2.8924 (13)	H15C···O6^iii^	2.47
N1···O4^ii^	2.9361 (13)	O6···H18A	2.66
O5···C8	2.9834 (12)	O6···H18B	2.58
O2···H12	2.44	N2···N1^iv^	3.0282 (14)
H16A···O2^i^	2.62	H1B···N2^iv^	2.152 (16)
O3···H10	2.4456	C7···C13	3.3501 (15)
H10···O4^iii^	2.57	C5···H9	2.67
O4···H16B	2.20	C7···H1B	2.616 (16)
H1A···O4^ii^	2.112 (16)	C14···H16B	2.70
O4···H15A	2.54	H4···H13	2.36
			
C2—O1—C6	120.07 (8)	C11—C10—H10	120.7
C14—O5—C15	115.20 (8)	C9—C10—H10	120.7
C17—O7—C18	115.07 (8)	C10—C11—C12	122.61 (9)
C2—N1—H1A	117.3 (10)	C10—C11—N3	118.87 (9)
C2—N1—H1B	118.5 (10)	C12—C11—N3	118.52 (9)
H1A—N1—H1B	119.2 (14)	C13—C12—C11	118.05 (9)
O3—N3—O2	123.95 (9)	C13—C12—H12	121.0
O3—N3—C11	118.11 (9)	C11—C12—H12	121.0
O2—N3—C11	117.94 (8)	C12—C13—C8	120.94 (9)
N1—C2—C3	127.86 (10)	C12—C13—H13	119.5
N1—C2—O1	110.58 (9)	C8—C13—H13	119.5
C3—C2—O1	121.46 (9)	O4—C14—O5	122.65 (9)
C2—C3—C7	119.33 (9)	O4—C14—C5	126.85 (10)
C2—C3—C4	122.70 (9)	O5—C14—C5	110.49 (8)
C7—C3—C4	117.77 (9)	O5—C15—H15A	109.5
C5—C4—C3	109.69 (8)	O5—C15—H15B	109.5
C5—C4—C8	111.99 (8)	H15A—C15—H15B	109.5
C3—C4—C8	109.73 (8)	O5—C15—H15C	109.5
C5—C4—H4	108.5	H15A—C15—H15C	109.5
C3—C4—H4	108.5	H15B—C15—H15C	109.5
C8—C4—H4	108.5	C6—C16—C17	110.25 (8)
C6—C5—C14	121.02 (9)	C6—C16—H16A	109.6
C6—C5—C4	122.16 (9)	C17—C16—H16A	109.6
C14—C5—C4	116.79 (9)	C6—C16—H16B	109.6
C5—C6—O1	122.47 (9)	C17—C16—H16B	109.6
C5—C6—C16	129.65 (9)	H16A—C16—H16B	108.1
O1—C6—C16	107.84 (8)	O6—C17—O7	124.87 (10)
N2—C7—C3	177.84 (11)	O6—C17—C16	125.10 (9)
C13—C8—C9	119.51 (9)	O7—C17—C16	110.03 (8)
C13—C8—C4	119.14 (8)	O7—C18—H18A	109.5
C9—C8—C4	121.25 (9)	O7—C18—H18B	109.5
C10—C9—C8	120.37 (9)	H18A—C18—H18B	109.5
C10—C9—H9	119.8	O7—C18—H18C	109.5
C8—C9—H9	119.8	H18A—C18—H18C	109.5
C11—C10—C9	118.51 (9)	H18B—C18—H18C	109.5
			
C6—O1—C2—N1	−172.01 (8)	C4—C8—C9—C10	175.54 (9)
C6—O1—C2—C3	4.55 (14)	C8—C9—C10—C11	0.07 (15)
N1—C2—C3—C7	−4.60 (16)	C9—C10—C11—C12	0.57 (15)
O1—C2—C3—C7	179.47 (9)	C9—C10—C11—N3	−178.79 (9)
N1—C2—C3—C4	−179.38 (9)	O3—N3—C11—C10	−12.19 (14)
O1—C2—C3—C4	4.70 (14)	O2—N3—C11—C10	167.63 (9)
C2—C3—C4—C5	−12.33 (13)	O3—N3—C11—C12	168.42 (9)
C7—C3—C4—C5	172.82 (8)	O2—N3—C11—C12	−11.75 (13)
C2—C3—C4—C8	111.09 (10)	C10—C11—C12—C13	−0.46 (15)
C7—C3—C4—C8	−63.76 (11)	N3—C11—C12—C13	178.90 (9)
C3—C4—C5—C6	12.41 (13)	C11—C12—C13—C8	−0.29 (15)
C8—C4—C5—C6	−109.67 (10)	C9—C8—C13—C12	0.91 (15)
C3—C4—C5—C14	−169.57 (8)	C4—C8—C13—C12	−175.50 (9)
C8—C4—C5—C14	68.34 (10)	C15—O5—C14—O4	1.14 (13)
C14—C5—C6—O1	177.07 (8)	C15—O5—C14—C5	179.93 (8)
C4—C5—C6—O1	−5.00 (15)	C6—C5—C14—O4	5.60 (16)
C14—C5—C6—C16	−5.52 (16)	C4—C5—C14—O4	−172.44 (9)
C4—C5—C6—C16	172.42 (9)	C6—C5—C14—O5	−173.13 (9)
C2—O1—C6—C5	−4.43 (14)	C4—C5—C14—O5	8.83 (12)
C2—O1—C6—C16	177.66 (8)	C5—C6—C16—C17	−99.61 (12)
C5—C4—C8—C13	−144.02 (9)	O1—C6—C16—C17	78.09 (10)
C3—C4—C8—C13	93.92 (10)	C18—O7—C17—O6	−2.19 (15)
C5—C4—C8—C9	39.64 (12)	C18—O7—C17—C16	178.38 (8)
C3—C4—C8—C9	−82.42 (11)	C6—C16—C17—O6	8.59 (15)
C13—C8—C9—C10	−0.79 (15)	C6—C16—C17—O7	−171.99 (8)

Symmetry codes: (i) *x*, *y*−1, *z*; (ii) *x*, −*y*+1/2, *z*+1/2; (iii) −*x*+1, −*y*+1, −*z*+1; (iv) −*x*+2, −*y*+1, −*z*+2.

### Methyl 6-amino-5-cyano-2-(2-methoxy-2-oxoethyl)-4-(4-nitrophenyl)-4H-pyran-3-carboxylate Hydrogen-bond geometry (Å, º)

**Table:**

*D*—H···*A*	*D*—H	H···*A*	*D*···*A*	*D*—H···*A*
N1—H1*A*···O4^ii^	0.887 (16)	2.112 (16)	2.9362 (12)	154.1 (13)
N1—H1*B*···N2^iv^	0.892 (17)	2.152 (17)	3.0283 (14)	167.2 (14)

Symmetry codes: (ii) *x*, −*y*+1/2, *z*+1/2; (iv) −*x*+2, −*y*+1, −*z*+2.
